# Junior Medical Officers’ knowledge of advance care directives and substitute decision making for people without decision making capacity: a cross sectional survey

**DOI:** 10.1186/s12910-022-00813-9

**Published:** 2022-07-18

**Authors:** Jamie Bryant, Amy Waller, Alison Bowman, Robert Pickles, Carolyn Hullick, Emma Price, Ben White, Lindy Willmott, Anne Knight, Mary-Ann Ryall, Rob Sanson-Fisher

**Affiliations:** 1 Health Behaviour Research Collaborative, University of Newcastle, Callaghan, Australia; 2 School of Medicine and Public Health, College of Health, Medicine and Wellbeing, University of Newcastle, Callaghan, NSW Australia; 3 Priority Research Centre for Health Behaviour, University of Newcastle, Callaghan, NSW Australia; 4 Equity in Health and Wellbeing Program, Hunter Medical Research Institute, New Lambton Heights, NSW Australia; 5 Belmont Hospital, Hunter New England Local Health District, Newcastle, NSW Australia; 6 John Hunter Hospital, Hunter New England Local Health District, Newcastle, NSW Australia; 7 Australian Centre for Health Law Research, Queensland University of Technology, Brisbane, QLD Australia; 8 Manning Education Centre, University of Newcastle Department of Rural Health, 69a High St, Taree, NSW Australia; 9 Wyong Hospital, Central Coast Local Health District, Gosford, NSW Australia

**Keywords:** Advance care directives, Advance care planning, Junior doctors, Knowledge

## Abstract

**Background:**

For the benefits of advance care planning to be realised during a hospital admission, the treating team must have accurate knowledge of the law pertaining to implementation of advance care directives (ACDs) and substitute decision making.

**Aims:**

To determine in a sample of Junior Medical Officers (JMOs): (1) knowledge of the correct order to approach people as substitute decision makers if a patient does not have capacity to consent to treatment; (2) knowledge of the legal validity of ACDs when making healthcare decisions for persons without capacity to consent to treatment, including the characteristics associated with higher knowledge; and (3) barriers to enacting ACDs.

**Methods:**

A cross-sectional survey was conducted at five public hospitals in New South Wales, Australia. Interns, residents, registrars, and trainees on clinical rotation during the recruitment period were eligible to participate. Consenting participants completed an anonymous pen-and-paper survey.

**Results:**

A total of 118 JMOs completed a survey (36% return rate). Fifty-five percent of participants were female and 56.8% were aged 20–29 years. Seventy-five percent of JMOs correctly identified a Guardian as the first person to approach if a patient did not have decision-making capacity, and 74% correctly identified a person’s spouse or partner as the next person to approach. Only 16.5% identified all four persons in the correct order, and 13.5% did not identify any in the correct order. The mean number of correct responses to the questions assessing knowledge of the legal validity of ACDs was 2.6 (SD = 1.1) out of a possible score of 6. Only 28 participants (23.7%) correctly answered four or more knowledge statements correctly. None of the explored variables were significantly associated with higher knowledge of the legal validity of ACDs. Uncertainty about the currency of ACDs and uncertainty about the legal implications of relying on an ACD when a patient’s family or substitute decision maker disagree with it were the main barriers to enacting ACDs.

**Conclusion:**

JMOs knowledge of the legal validity of ACDs for persons without decision making capacity and the substitute decision making hierarchy is limited. There is a clear need for targeted education and training to improve knowledge in this area for this cohort.

## Background

Advance care planning (ACP) is the process of discussing and documenting a person’s values, beliefs and preferences about future health needs to guide decision-making about care if an individual does not have the capacity or ability to communicate this information themselves [1, 2]. Research indicates that benefits of ACP include higher quality end-of-life care, greater compliance with end-of-life wishes, reduced health care costs and reduction of stress, anxiety, and depression in surviving relatives [3–5].

The overarching goal of ACP is to ensure that people receive medical care that is consistent with their values, goals, and preferences. ACP includes a range of key activities, ranging from informal conversations about preferences and goals of care, to formal activities such as the completion of legal written documents, such as Advance Care Directives (ACD) [6]. An ACD is a specific type of ACP tool completed by an adult with decision-making capacity. An ACD may include: a nominated person or persons to make medical decisions for that adult (i.e. a substitute decision-maker[SDM]); details of the person’s values, life goals and preferred outcomes and treatments, and information about the care that is preferred or would be refused in the event of a life-threatening illness or injury [1]. ACP can be undertaken by anyone, but it is particularly relevant for those who have been diagnosed with a serious illness.

The laws pertaining to ACDs vary by state across Australia [7]. For instance, in New South Wales (NSW) an instructional ACD is recognised by common law rather than legislation and can be in made in writing or spoken. For such an ACD to be valid: (1) the person making it must have had capacity (decision-making ability) at the time of drafting; and (2) it must be made freely and voluntarily [7]. For an ACD to be binding on doctors, it must also have been intended to operate in the circumstances that have later arisen [7]. There may be doubt about this, for example, where an ACD gives only vague instructions about treatment or there are doubts about currency (e.g., there is evidence the person later changed their mind). Health professionals and ‘persons responsible’ (i.e. someone who is legally able to make medical and dental decisions on behalf of another person who lacks the capacity to give their own consent to treatment [8]), cannot override a valid ACD [1]. NSW law, as is commonly the case in Western jurisdictions, also recognises the legal appointment of a SDM both by the person in advance of them losing capacity and by a tribunal [7].

Most medical practitioners state they would use ACP instruments, such as ACDs, to guide treatment for people who lack capacity to consent to treatment [9, 10]. However, for the benefits of ACP to be fully realised during a hospital admission, all members of the treating team must have accurate knowledge of the law pertaining to ACP, including the legal validity of ACDs, when ACDs can and should be applied. A large study exploring knowledge of medical practitioners across three Australian states identified major gaps in knowledge about the law with respect to withholding and withdrawing life-sustaining treatment from adults who lack capacity, even among medical specialists typically involved in end-of-life decision-making [11]. The findings of this study suggest that strategies to improve the legal knowledge of medical practitioners may be required to ensure compliance with the law [11]. To date however, no research has been conducted to assess the knowledge of Junior Medical Officers’ (JMOs) of the legal validity of ACDs. This is an important gap in the literature. JMOs include trainees, registrars, residents and/or interns. In the course of their work, JMOs are often required to treat patients who do not have capacity to consent to treatment, such as those with dementia, to treat patients presenting to hospital with ACDs, and to initiate conversations about completing ACDs. Understanding the knowledge of JMOs pertaining to ACP law, and their perceptions of barriers to using ACDs in clinical practice, is critical to ensuring JMOs are compliant with the law and facilitating patient choices in care.

This study therefore aimed to determine JMOs:

1. Knowledge of the correct order in which people should be approached to be a substitute decision maker if a patient does not have capacity to consent to their own treatment.
2. Knowledge of the legal validity of ACDs when making healthcare decisions for persons without capacity to consent to treatment, including the characteristics associated with higher knowledge.
3. Perceptions of the barriers to enacting ACDs in the hospital setting.

## Methods

### Design and setting

A cross-sectional survey conducted with JMOs from five public hospitals in New South Wales, Australia.

### Eligibility

JMOs including interns, residents, registrars, and trainees on clinical rotation at participating hospitals during the recruitment period were eligible to participate.

### Recruitment

Eligible participants were approached to participate between August 2018 and May 2019 by co-researchers or senior clinical staff during scheduled training sessions, orientation days and/or at ward rounds. Participants were given a verbal overview of the research, then provided with a study recruitment package which included a paper copy of the survey, a detailed Participant Information Sheet, and a return reply-paid envelope.

### Data collection

Consenting participants completed an anonymous 64 item pen-and-paper survey. Completion of the survey was taken as implied consent. Participants either completed the survey during pre-scheduled education sessions, during shift, or in their own time. Participants could return their completed survey to a secure box located in a common room, or mail back to the research team using the provided reply-paid envelope.

### Measures

A draft survey was developed based on a literature review of legal aspects of ACP practices, and discussion with senior experienced clinicians and lawyers. The draft survey was reviewed by a panel of experts including behavioural scientists, lawyers, emergency physicians, general physicians and nurses and items refined based on feedback. The survey was pilot tested for acceptability, relevance, and clarity of the items with a sample of five JMOs and refined based on feedback.

#### Person responsible hierarchy

Under the *Guardianship Act 1987* (NSW), a ‘person responsible’ is the person who can consent to medical and dental treatment for a person who is unable to provide consent themselves. There is a hierarchy in which a person responsible should be approached by a treating health practitioner to obtain substitute consent to treatment is as follows: (1) Guardian or Enduring Guardian; (2) Spouse (including de facto spouse or same sex partner) who has a close and continuing relationship with the patient; (3) Unpaid carer or person who arranges care regularly for the patient; (4) Close friend or relative. Participants were presented with information about the *Guardianship Act 1987* (NSW). Participants were then asked to rank from 1 to 4 the order in which individuals listed should be approached to be a patient’s person responsible if they did not have capacity to consent to their own medical treatment (1 being the 1^st^ person approached, 4 being the last person approached).

#### Knowledge of legal validity of ACDs

Participants were presented with six statements regarding the legal validity of advance care directives (3 questions), the legal authority of Enduring Guardians and SDMs (2 questions) and treatment provision to patients without decision making capacity (1 question). Statements were derived from items previously used with doctors in three Australian states [12]. Participants were asked to respond ‘true’, ‘false’, or ‘I don’t know’ for each item.

#### Barriers to enacting ACDs in hospital

The following definition of an ACD was provided “*An Advance Care Directive is a legally binding document that can include:* (1) *who a patient wants to make medical decisions for them if they are unable (a substitute-decision maker);* (2) *what is important to a patient (e.g., values, life goals and preferred outcomes); and* (3) *the medical care a patient would accept or refuse. An Advance Care Directive is different to an Adult Resuscitation Plan*.” Participants were presented with eight items and asked to rate the extent of their agreement that each was a barrier to enacting ACDs in hospital on a four-point Likert scale from strongly agree to strongly disagree.

#### Socio-demographic characteristics and clinical experience

Participants self-reported their: gender; age; where their medical degree was obtained; years’ experience as a doctor; clinical rotations completed; whether they were enrolled in a specialist training program; whether they had ever provided care to a patient with an ACD and whether they had completed post-graduate courses or training about ACP.

### Statistical analysis

Data were analysed using SAS v9.4 [13]. Data for each item were summarised using descriptive statistics. Results for the ranking of the hierarchy of persons responsible were summarised in a distribution table of answers (ranks) for each question. For each knowledge item, frequencies, and percentages of correctly answered items were calculated. A correct response was given a score of one. Incorrect answers, and those marked as ‘I don’t know’, received a score of zero. A total knowledge score for each participant was created by summing the number of correct answers across the six knowledge items (range 0–6), and a mean knowledge score also calculated. Socio-demographic and clinical experience characteristics associated with total knowledge score were examined using regression analyses for complete cases.

## Results

A total of 328 surveys were distributed to JMOs of which 118 surveys were returned (36% of eligible participants). Demographic details of participants are presented in Table 1. Slightly more of the participants were female (n = 65, 55.1%), aged 20–29 years (n = 67, 56.8%) and had four or more years of post-graduate training (n = 46, 39%). Only 11.9% (n = 14) of participants had received post-graduate training about advance care planning.

**Table 1:** Participant demographic characteristics (n = 118)

Variable	Category	N (%)
Gender	Male	50 (42.4%)
	Female	65 (55.1%)
	Missing	3 (2.5%)
Age	20–29	67 (56.8%)
	30–39	41 (34.7%)
	40–49	4 (3.4%)
	50 or over	3 (2.5%)
	Missing	3 (2.5%)
Medical degree obtained in Australia?	Yes	87 (73.7%)
	No	27 (22.9%)
	Missing	4 (3.4%)
Number of years’ experience	Post graduate year 1	18 (15.3%)
	Post graduate year 2	42 (35.6%)
	Post graduate year 3	9 (7.6%)
	Post graduate year 4 or greater	46 (39.0%)
	Missing	3 (2.5%)
Enrolled in specialist training program	Yes	51 (43.2%)
	No	59 (50.0%)
	Missing	8 (6.8%)
Post-graduate training about advance care planning	Yes	14 (11.9%)
	No	99 (83.9%)
	Missing	5 (4.2%)

### Knowledge of person responsible hierarchy

Seventy-five percent (n = 87) of JMOs correctly identified a Guardian as the first person to approach in the event a patient did not have decision-making capacity, 74% (n = 85) correctly identified a person’s spouse as the next person to approach, 22% (n = 25) correctly identified an unpaid carer as the third person to approach, and 24% (n = 28) correctly identified a friend or relative as the fourth person to approach. Overall, 74% of the sample (n = 85) correctly identified the first and second people responsible. However only 16.5% (n = 19) identified all four persons responsible in the correct order, and 13.5% (n = 21) did not identify any of the persons responsible in the correct order.

### Knowledge of ACDs

**Fig. 1:**
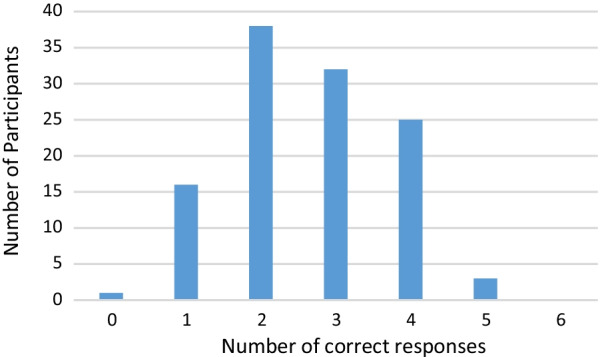
Distribution of the number of correct responses for the knowledge of advance care (directive) law questions (n = 115*)

Figure 1 shows the distribution of the number of correct responses for the knowledge of advance care planning law questions. Overall, the mean number of correct responses was 2.6 (SD = 1.1) out of a possible score of 6. Only 28 participants (23.7%) correctly answered four or more of the statements correctly, with 87 (73.7%) answering three or fewer correctly. No participants answered all six statements correctly.

Table 2 shows the results of the logistic regression looking at factors associated with higher knowledge. There were 107 complete cases for the multivariable regression. None of the explored variables were significantly associated with higher knowledge about the legal validity of ACDs.

**Table 2:** Logistic regression examining the demographic characteristics associated with answering ≥ 4 statements correctly (n = 107)

	Unadjusted		Adjusted	
	Estimate (95% CI)	*P* value	Estimate (95% CI)	*P* value
*Gender*				
Female	0.01 (− 0.39, 0.41)	0.9575	0.11 (− 0.32, 0.53)	0.6212
Male	Ref		Ref	
*Age*				
20–29	Ref	0.8676	Ref	0.3839
30 or more	0.03 (− 0.36, 0.43)		− 0.22 (− 0.72, 0.28)	
*Medical degree*				
Australia	− 0.09 (− 0.56, 0.37)	0.6906	− 0.04 (− 0.59, 0.50)	0.8774
Overseas	Ref		Ref	
*Years post-graduate*				
2 or less	Ref	0.2530	Ref	0.3695
3 or more	0.23 (− 0.16, 0.62)		0.26 (− 0.31, 0.83)	
*Enrolled in specialist training*				
Yes	0.17 (− 0.23, 0.56)	0.4113	0.03 (− 0.49, 0.54)	0.9234
No	Ref		Ref	
*Postgraduate training in ACP*				
Yes	0.19 (− 0.41, 0.79)	0.5362	0.14 (− 0.46, 0.74)	0.6485
No	Ref		Ref	

### Barriers to enacting ACDs

Table 3 shows barriers to enacting ACDs. The most frequently reported barriers to enacting ACDs were uncertainty about the currency of the ACD, and uncertainty about the legal implications of enacting when a patient’s family or SDM disagree with the ACD (83% and 82% agreement respectively). More than 70% of participants also agreed that difficulty accessing ACDs, poor knowledge among doctors about what constitutes a legally binding ACD, and lack of detail and specificity within ACDs were barriers to implementing ACDs.

**Table 3:** Barriers to enacting ACDs (n = 118*)

	Strongly agree	Agree	Disagree	Strongly disagree
Uncertainty about the currency of the advance care directive (i.e., does it represent the patient’s current values and wishes?)	20 (17.5%)	78 (68.4%)	15 (13.2%)	1 (0.9%)
Uncertainty about the legal implications of enacting when a patient’s family or substitute decision maker disagree with the advance care directive	33 (29.2%)	64 (56.6%)	15 (13.3%)	1 (0.9%)
Difficulty accessing the advance care directive when treatment decisions need to be made	41 (36%)	51 (44.7%)	19 (16.7%)	3 (2.6%)
Poor knowledge among doctors about what constitutes a legally binding advance care directive	23 (20.4%)	64 (56.6%)	24 (21.2%)	2 (1.8%)
Poor knowledge among doctors about the circumstances in which an advance care directive should be used	18 (15.8%)	48 (42.1%)	46 (40.4%)	2 (1.8%)
Lack of detail and specificity within the advance care directive to meaningfully guide decision making	24 (21.1%)	59 (51.8%)	30 (26.3%)	1 (0.9%)
Use of vague language in the advance care directive, which makes it difficult to use it to meaningfully guide decision making	28 (24.6%)	45 (39.5%)	40 (35.1%)	1 (0.9%)
Difficulty identifying a patient’s substitute decision maker	14 (12.3%)	59 (51.8%)	40 (35.1%)	1 (0.9%)

*Row totals do not sum to 118 due to missing variables

## Discussion

To our knowledge, this is the first Australian study to focus on knowledge of junior doctors about the implementation of ACDs. Our findings demonstrate there are critical gaps in the knowledge of JMOs about substitute decision making and the legal considerations of implementing ACDs for patients without capacity to consent.

While almost three quarters of participants correctly identified the first and second people to approach for substitute decision making in the event a person is unable to make their own treatment decisions, there was uncertainty about who should be approached if an individual did not have an appointed guardian, or a spouse or partner. Few participants identified all four persons responsible in the correct order. In the event an individual does not have capacity, healthcare providers have an obligation to consult with the person highest on the hierarchy to make treatment decisions [14]. Accurate knowledge of who the legally authorised SDM is for a patient without capacity, and the types of treatment they can and cannot consent to, is therefore critical to ensure compliance with the law and respect for patient wishes. The low rate of knowledge about who to approach could lead to acting on a decision of a person who has no legal power to decide or the giving or withholding of inappropriate treatment against the articulated wishes of the patient, infringing their rights, and potentially leading to legal consequences for healthcare providers.

Participants demonstrated overall limited knowledge of the validity of ACDs when making healthcare decisions for people without decision making capacity. For the six statements presented, no participants answered all correctly, and only 23% of participants answered four or more statements correctly. A previous national study found similar gaps in legal knowledge of medical practitioners across seven specialties using a similar instrument with minor variations for local state laws [11], finding a mean knowledge score of 2.97 compared with the mean score of 2.6 for JMOs. This data suggests that senior doctors experience similar knowledge gaps and barriers to their junior staff and may not be well placed to provide advice and further training in this area. Lack of legal knowledge among JMOs is particularly significant given that more than three quarters of participants agreed that poor knowledge about what constitutes a legally binding ACD was a barrier to implementation in the hospital setting, and a further 57% agreed that poor knowledge among doctors about the circumstances in which ACDs should be used was a barrier to ACD implementation. This aligns with the main barriers perceived to impact implementation of ACDs found in qualitative work completed with more experienced doctors in Victoria across specialties, which found that concerns about the validity and currency of ACDs, subjective terminology, and family opposition to implementation of ACDs were common barriers to care [15]. Specialists are seen as a source of information about end-of-life law by junior doctors. In a survey of medical specialists, 41% of respondents reported they were often or very often asked by interns, residents, and registrars about the law pertaining to withholding or withdrawing life-sustaining treatment [16].

These results highlight the need for targeted training and resources for junior doctors in the legal aspects of ACDs and substitute decision-making, especially in the context of the complexity of the law and prognostic uncertainty. In this sample of JMOs, 89.3% reported not completing any post-graduate training in ACP. Training should support doctors’ knowledge and confidence in understanding relevant law and the civil and criminal consequences of not doing so. A recent systematic review [17] that critically examined ten ACP training programs for healthcare professionals found that all had positive impacts on outcomes including knowledge, attitudes, skills, and comfort of participants in discussing issues related to end of life decision-making. This review recommends small group discussions, communication skills training workshops, the use of role-play and programs including the ‘Conversation Starter Kit’ and the ‘Respecting Patient Choices Program’ as effective models for overcoming knowledge gaps. Short interactive education programs and workshops have been demonstrated to improve student and doctors knowledge and confidence in engaging in ACP, and their performance in simulated ACP activities [18, 19]. Prioritising the use of available training in end-of-life law by JMOs, as well as ongoing training to further support skill development, is critical to improving the provision of high-quality patient-focused care. Other challenges of appropriately enacting ACDs and involving SDMs in decision making should also be acknowledged. ACP is complex and while clinician knowledge is necessary, it is not on its own sufficient to ensure the provision of high-quality care for people without decision making capacity in clinical settings where multiple and competing demands impact on practice. Patient and systems-level barriers and enablers to the implementation of ACDs, and how these factors interact, also need to be considered [20].

### Limitations

Participants were drawn from five hospitals in New South Wales. However, the sample size was small, and the response rate limits the generalisability of the findings. Further, each Australian state and territory has some variation in laws regarding the ‘person responsible’ hierarchy, therefore these findings may not be directly applicable beyond New South Wales. We also did not assess how long JMOs had been working in NSW. Given that laws pertaining to ACDs vary by state across Australia, it is possible that a lack of familiarity with the laws in NSW may have contributed to low knowledge scores.

### Conclusion

JMOs knowledge of the legal validity of ACDs for persons without capacity to consent for treatment and the substitute decision making hierarchy is limited. There is a clear need for targeted education and training to improve knowledge in this area for this cohort.

## Data Availability

The datasets used and/or analysed for this study are available from the corresponding author upon reasonable request.

## Abbreviations

JMO: Junior Medical Officer
ACP: Advance care planning
ACD: Advance care directive
SDM: Substitute decision maker
